# Neonatal hair profiling reveals a metabolic phenotype of monochorionic twins with selective intrauterine growth restriction and abnormal umbilical artery flow

**DOI:** 10.1186/s10020-020-00160-8

**Published:** 2020-05-01

**Authors:** Jing Yang, Yuan Wei, Hongbo Qi, Nanlin Yin, Yang Yang, Zailing Li, Lili Xu, Xueju Wang, Pengbo Yuan, Luyao Li, Ting-Li Han, Yangyu Zhao

**Affiliations:** 1 Department of Obstetrics & Gynecology, Peking University Third Hospital, No.49 North HuaYuan Road, HaiDian District, Beijing, 100191 China; 2 Department of Obstetrics, The First Affiliated Hospital of Chongqing Medical University, Chongqing, 400716 China; 3 State Key Laboratory of Maternal and Fetal Medicine of Chongqing Municipality, The First Affiliated Hospital of Chongqing Medical University, Chongqing, 400716 China; 4 Department of Pediatrics, Peking University Third Hospital, Beijing, 100191 China; 5 Liggins Institute, University of Auckland, Auckland, 1142 New Zealand

**Keywords:** Monochorionic diamniotic (MCDA) twins, Selective intrauterine growth restriction (sIUGR), Umbilical artery flow (UAF), Hair, Metabolomics, And fetal growth velocity

## Abstract

**Background:**

Selective intrauterine fetal growth restriction (sIUGR) in monochorionic diamniotic twins, especially types 2&3 with abnormal umbilical artery Doppler, results in increased risk of fetal/perinatal mortality and postnatal disability. We investigate whether the hair metabolome profiles of neonates were associated with the pathophysiological differences across the different clinical forms of sIUGR in twins.

**Methods:**

Hair samples were collected at delivery from 10 pairs of type 1 sIUGR twins, 8 pairs of types 2&3 sIUGR twins, and 11 pairs of twins without sIUGR. The hair metabolome was characterized using gas chromatography-mass spectrometry.

**Results:**

Our results demonstrated that the hair metabolite profiles of the different sIUGR subclinical forms were associated with the averaged fetal growth rate after 28 weeks of gestation but not with birthweight. The hair profiles were capable of discriminating type2&3 sIUGR twins from twins without sIUGR. In particular, the metabolites 2-aminobutyric acid, cysteine, alanine, and tyrosine all displayed areas under the receiver operating characteristic curve were above 0.9. The metabolic pathway analysis highlighted the associations of sIUGR twins with abnormal umbilical artery flow with increased metabolites from a nutrient depletion pathway, glutathione metabolism, and nerve development.

**Conclusion:**

This study offers novel insight into the severity of intrauterine ischemia and hypoxia for T2&3 sIUGR twins, through evaluation of the neonatal hair metabolome.

## Background

The incidence of twin pregnancies is rising significantly. The main reasons for the noticeable rise in twin pregnancies is the increase in delayed childbirth resulting in advanced maternal age at conception, and increased use of assisted reproduction techniques (Multiple pregnancy 2011). Compared with singletons, twin pregnancies suffer from a greater incidence of maternal and fetal complications (ACOG Practice Bulletin No. 144 2014; DeJesus Allison et al. 2012). The most common complications experienced in twin pregnancies are gestational diabetes mellitus (GDM; 20.4%) (McGrath et al. 2017), pre-eclampsia (10%) (Fox et al. 2014), and preterm birth (50%) (Hediger et al. 2005). There is an estimated five-fold increase in fetal mortality and a seven-fold increase in neonatal death compared with singletons^2^, which is primarily due to complications of prematurity. Unequal sharing of the placental and vascular communications between twins leads to a significantly higher risk of various complications in monochorionic (MC) twins compared to dichorionic (DC) twins (Hack et al. 2008a; Southwest Thames Obstetric Research C 2012).

Selective intrauterine fetal growth restriction (sIUGR) occurs in 10–15% of MC twin pregnancies. The clinical classification of sIUGR is according to Doppler waveform of end-diastolic velocity in umbilical artery flow (UAF), diagnosed by ultrasound (Gratacos et al. 2007). Type 1 is sIUGR with normal UAF; Type 2 is sIUGR with absence/reverse UAF; Type 3 is sIUGR with intermittent absence/reverse UAF. Twin pregnancies with Type 1 sIUGR have higher than 90% survival rate and can be managed expectantly (Valsky et al. 2010), while types 2 and 3 (T2&3) sIUGR are associated with a high risk of perinatal mortality of the growth restricted twin (Gratacós et al. 2004). The management of T2&3 sIUGR have proven to be clinically challenging due to the inter-dependence of twin connection through the placental vasculature, as well as complex issues such as selective feticide, parental choice, and the high incidence of early preterm delivery^10^.

There are an increasing number of studies utilizing metabolomic profiling to uncover the pathophysiology of sIUGR twin pregnancies. Clinical metabolomic profiling is a powerful top-down system biology approach to investigate the low molecular weight biochemicals (metabolites) present in a cell, tissue, or organism. Although metabolomic studies of MCDA twins with sIUGR have occurred in cord plasma and placenta (Wang et al. 2018; Cosmi et al. 2013), the metabolic profiles of complex biological fluids and tissues are highly dynamic. In the view that most sIUGR twins are established before 26 weeks’ gestation, a long ‘latency period’ exists between diagnosis and delivery. There is a necessity to establish accurate strategies for the assessment of longitudinal pathophysiology associated with sIUGR in MC twins with abnormal UAF Doppler.

Hair offers a unique advantage over other biological tissues/matrices, most notably the potential to retrospectively reconstruct exposures over sequential periods of weeks to months across the length of the hair strand. Hair becomes a particularly useful source of biological information over a period of time because of its highly stable structure which retains endogenous and exogenous compounds (Eastman et al. 2013). Especially, hair follicles begin to grow at around 10 weeks of gestation and cover the entire scalp of the fetus by 20 weeks’ gestation (Gareri and Koren 2010; Furdon and Clark 2003). Nevertheless, follicles then undertake their first life cycle and the earliest development of fetal hair will not occur until 24 to 28 weeks of gestation (Gareri and Koren 2010; Paus and Cotsarelis 1999). This indicates that fetal hair harvested at delivery is likely to reflect the metabolic activity post 28 gestational weeks. In addition, hair collected immediately after birth is not susceptible to contamination from the external environment. Therefore, hair has great potential to reflect the longitudinal exposures incurred under the detrimental intrauterine conditions experienced by sIUGR twins. Therefore, the aim of this study is to analyse the neonatal hair metabolome coupled with ultrasound assessment to assess the longitudinal exposures incurred under the detrimental intrauterine conditions experienced by T1 sIUGR and T2&T3 sIUGR twins.

## Methods

### Study participants and exclusion criteria

This was a nested case-control study. Participants were recruited from an ongoing cohort study at the Peking University Third Hospital as part of the University Hospital Advanced Age Pregnant Cohort (clinicaltrials.gov Identifier: NCT03220750). A total of 142 MCDA twin pregnancies were enrolled in the cohort between September 2017 and December 2018. Participants with maternal chronic diseases, major fetal structural anomalies, other adverse twin pregnancy outcomes, and those lost to follow-up at birth were excluded from participation in this study (Supplementary Figure 1). Twins with abnormal karyotypes were also excluded, as determined by non-invasive chromosome-selective sequencing of fetal cell-free DNA in maternal blood or amniocentesis in the second trimester. After these exclusions, 18 pairs of sIUGR twin pregnancies remained in our study, with a combination of 13 early and five late onset sIUGR diagnosed prior and after 24 weeks gestation, respectively. Among them, eight twin pregnancies were identified with types 2&3 sIUGR with abnormal UAF and 10 twin pregnancies were diagnosed with type 1 sIUGR with normal UAF. Moreover, 11 pairs of uncomplicated MCDA twin pregnancies matched to cases according to maternal age and BMI, were selected as a control group. Lastly, maternal and fetal clinical characteristics were measured and recorded within 24 h after delivery.

### Ultrasound assessment

In our hospital, all ultrasound examinations prior to 16 weeks’ gestation are performed by three different obstetric sonographers. After recruitment at 16 weeks’ of gestation, one certified twin specialist obstetric sonographer is assigned to perform both ultrasound examinations and record fetal biometry/Doppler indices on all recruited participants at least once every two weeks according to the guideline of the International Society of Ultrasound in Obstetrics and Gynecology (ISUOG) (Khalil et al. 2016).

### Determination of gestational age

The determination of gestational age for spontaneous conception was calculated using the first day of the female’s last menstrual period and this date was confirmed with the ultrasound measurement of the crown-rump length of the larger twin during 10–14 weeks’ gestation. Gestational age of pregnancies conceived by in-vitro fertilization were ascertained according to the embryonic age on the date of embryo transfer. The ultrasound assessment to determine gestational age was performed by three different obstetric sonographers.

### Determination of fetal growth rate

Fetal growth rate (grams/week) was determined by calculating weekly average growth of estimated fetal weight (EFW) from 28 gestational weeks until delivery using linear interpolation (Hediger et al. 2005). EFW was calculated using ultrasound measurements of biparietal diameter (BPD), head circumference (HC), abdominal circumference (AC), and femur length (FL) using the mathematical formula proposed by Hadlock et al. (Hadlock et al. 1985). Both ultrasound examinations and determination of fetal growth rate were completed by a fixed obstetric sonographer.

### Diagnosis of MCDA pregnancy and sIUGR twins

**Fig. 1:**
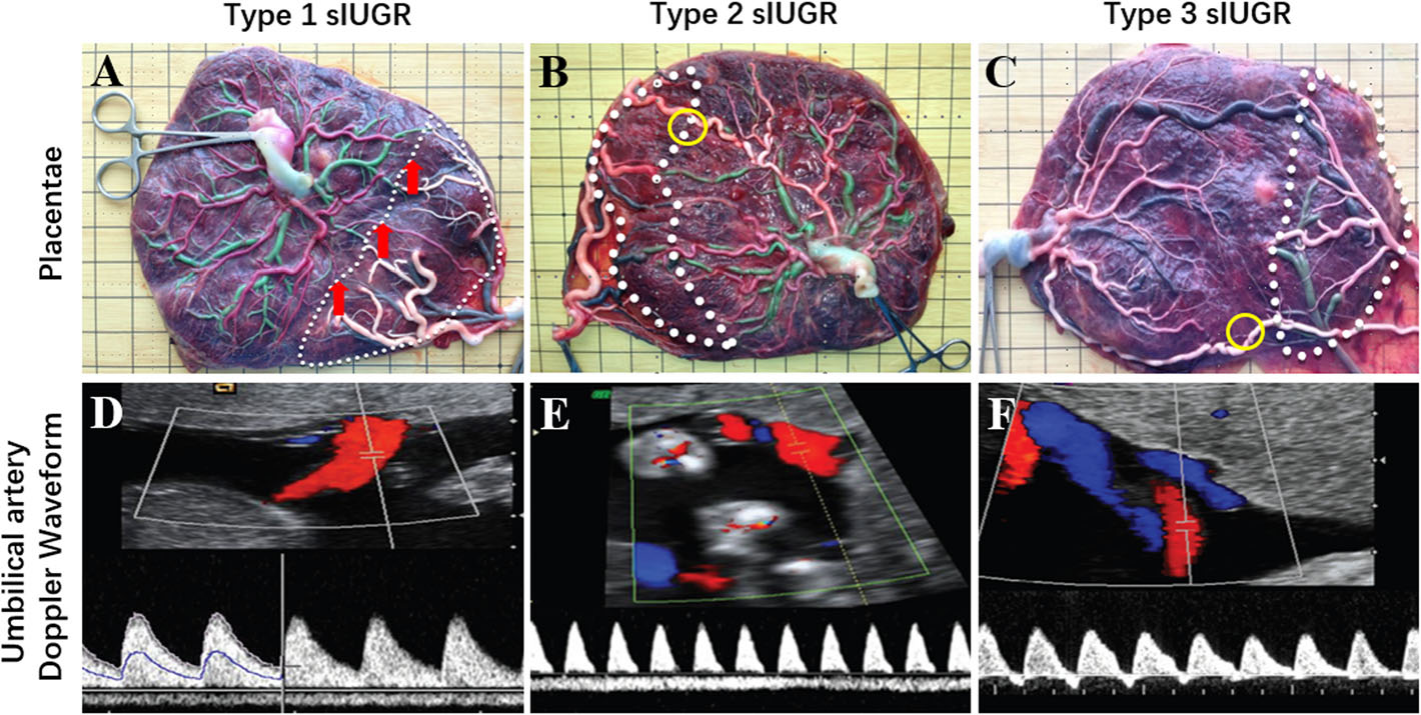
Classifications of type 1, type 2, and type 3 selective intrauterine growth restriction (T1& T2&T3 sIUGR) in monochorionic twins via placental sharing, velamentous cord insertion, anastomoses, and umbilical artery flow. The white dotted lines connect the smallest diameter vascular anastomoses points on the surface of the chorionic plate and divide the placenta into larger and smaller twin portions. The red regions in the **d**, **e**, and **f** indicate that the blood flow of umbilical artery directs toward the ultrasound probe. The blue regions in the **e**, **f** show that the direction of blood flow in the umbilical artery is opposite to the ultrasound probe. **a** T1 sIUGR placenta - placenta was infused with placental vascular dye injection. This displays unequal placental sharing and discordance cord insertion as the features of sIUGR placenta, but vascular anastomoses remain similar to uncomplicated monochorionic twins. **b** T2 sIUGR placenta - large arterioarterial anastomoses (2 ≥ mm, yellow circle) are present in 18% of placenta. **c** T3 sIUGR placenta **-** large arterioarterial anastomoses (≥2 mm, yellow circle) are present in 98% of placenta. **d** In T1 sIUGR, the umbilical artery Doppler waveform has positive end-diastolic velocity in the umbilical artery. **e** In T2 sIUGR, there is absent or reversed end-diastolic flow (AREDF) in the umbilical artery. **f** In T3 sIGUR, there is a cyclical or intermittent pattern of AREDF in the umbilical artery

MCDA pregnancy diagnosed as monochorionic and diamniotic was determined via ultrasound in the following two periods: a) displaying a single gestational sac via ultrasound by 10 gestational weeks and b) presenting with T-sign of the intertwin membrane and absent twin peak (lambda) sign via ultrasound between 11 and 14 gestational weeks. A final pathologic evaluation of the placental features after delivery confirmed monochronic twins participated in this study. The diagnostic criteria for sIUGR was defined by at least one of the twin’s EFW being less than the 10th percentile for the corresponding gestational age and an intertwin EFW discordance greater than 25%. The sIUGR twins were then classified into type-1/2/3 sIUGR using Doppler velocimetry via ultrasound of the end-diastolic umbilical arteries as detailed in Fig. 1. The diagnosis of chorionicity, amnionicity, and type of sIUGR was determined by a fixed obstetric sonographer. Subsequently, two senior physicians with maternal-fetal medicine specialization decided treatment strategies for the sIUGR pregnancies.

### Hair collection

Immediately after twin delivery, hair samples were cut 0.5 cm proximal from the scalp of each twin with clean scissors and then stored at − 20 °C until the samples were processed. All hair samples underwent only a single freeze-thaw cycle before analysis.

### Hair sample preparation

Hair sample preparation was conducted according to the published protocol by Sulek et al with minor modifications (Sulek et al. 2014). All hair samples were randomised prior to sample preparation and weighed to 3.5 mg ± 0.5 mg. All weighed hair samples were washed with distilled water and methanol twice. Three internal standards, 20 μL of D4-alanine (Sigma, USA, 10 mM), D5-phenylalanine (Sigma, USA, 10 mM), and D2-tyrosine (Sigma, USA, 10 mM) were added to the hair samples and incubated with 1 ml potassium hydroxide (1 M) at 54 °C for 18 h. The hair extracts were then neutralized by adding 67 μL sulphuric acid (3 M). To precipitate the salt and protein, 1 ml of methanol was added to the hair extracts, followed by vortexing for 30 min, and centrifugation at 4000 g for 5 min. The 350 μL of supernatant was concentrated to dryness in a SpeedVac (Labconco, Kansas, USA) at 37 °C for 6 h and stored at − 20 °C prior to derivatization. Quality control (QC) samples were also prepared by combining a small portion of all hair extracts together and following the identical preparation steps to the samples.

### Chemical derivatization and gas chromatography-mass spectrometry (GC-MS) analysis

Dried hair extracts were resuspended in 200 μL of sodium hydroxide (1 M) and methyl chloroformate derivatization was performed to make the compounds more volatile for GC-MS analysis, based on the protocol published by Smart et al (Smart et al. 2010). All neonatal hair samples were analysed in a single batch and derivatized compounds were separated by a GC7890 chromatography system using a ZB-1701 GC capillary column. (30 m × 250 μm id × 0.15 μm with a 5 m guard column, Phenomenex) and analyzed by a MSD5975 mass spectrometer (Agilent, California, USA) with electron impact ionization via electron emission at 70 eV. The GC temperature program and MS parameters were set up according to the protocol described in Smart et al (Smart et al. 2010).

### Metabolite identification, data mining, and statistical analysis

Automated Mass Spectral Deconvolution & Identification System software was implemented for metabolite deconvolution. The compounds were identified by comparing the MS fragmentation patterns (mass-to-charge ratio and relative intensity of mass spectra to a reference ion) and respective GC retention time to an in-house MS library established using chemical standards. The remaining putative compounds were identified using a commercial NIST mass spectral library. The MassOmics XCMS R-based script was used to extract the relative concentration of the metabolites through the peak height of the most abundant fragmented ion mass. To improve quantitative robustness along with minimizing human and instrumental variability, the relative abundances of the identified compounds were normalized in the order of multiple internal standards (D4-alanine, D5-phenylalanine, or D2-tyrosine was selected to normalise each metabolite based on their correlation with metabolites in the QC samples), median centering-batch correction via nine QC samples (three QC samples per batch), and dried hair weight of the corresponding sample. Then, blank samples were used to subtract background contamination and any carryover from identified metabolites. Student’s *t*-test, non-parametric Mann-Whitney U test, Chi-square test, and Fisher’s exact test were performed in R to compare maternal and twin clinical characteristics. Prior to statistical analysis, the hair metabolite profiles were adjusted by log transformation and Pareto scaling because these combined scaling methods provided the best Gaussian distribution for this neonatal hair dataset. Partial least squares discriminant analysis (PLS-DA) with leave-one-out cross validation (LOOCV) was performed to compare the hair metabolome profiles between different twin groups via Metaboanalyst 3.0 package for R (Xia et al. 2015). Adjusted logistic regression was performed to account for confounding factors and false discovery rates were reported to account for multiple comparisons. The area under the receiver operating characteristic (ROC) curve of the relative concentration of hair metabolites was calculated using the pROC R-package (Robin et al. 2011) and multivariate ROC curves were constructed using a linear support vector machine model performed on the MetaboAnalyst website (https://www.metaboanalyst.ca). Generalized estimating equation (GEE) modeling was implemented to identify hair metabolites correlated with birthweight and growth rate discordances within twin pairs and between twin pairs. Metabolic pathway activity was estimated using the Pathway Activity Profiling (PAPi) R-algorithm (Aggio et al. 2010). The metabolic network was reconstructed based on the KEGG database through Cytoscape (Version 3.6.1). The intra- and inter-observer reliability of the ultrasound measurement was calculated using the Bland-Altman method (Bruin et al. 2019) via an R console, while the classifications of sIUGR were determined by the Kappa value calculated using SPSS software. The graphic illustrations of heatmaps, line graphs, and circos plots were created using ggplot2 and GOplot R-packages (Wickham 2009; Walter et al. 2015).

## Results

### The rationale underlying our study design

**Fig. 2:**
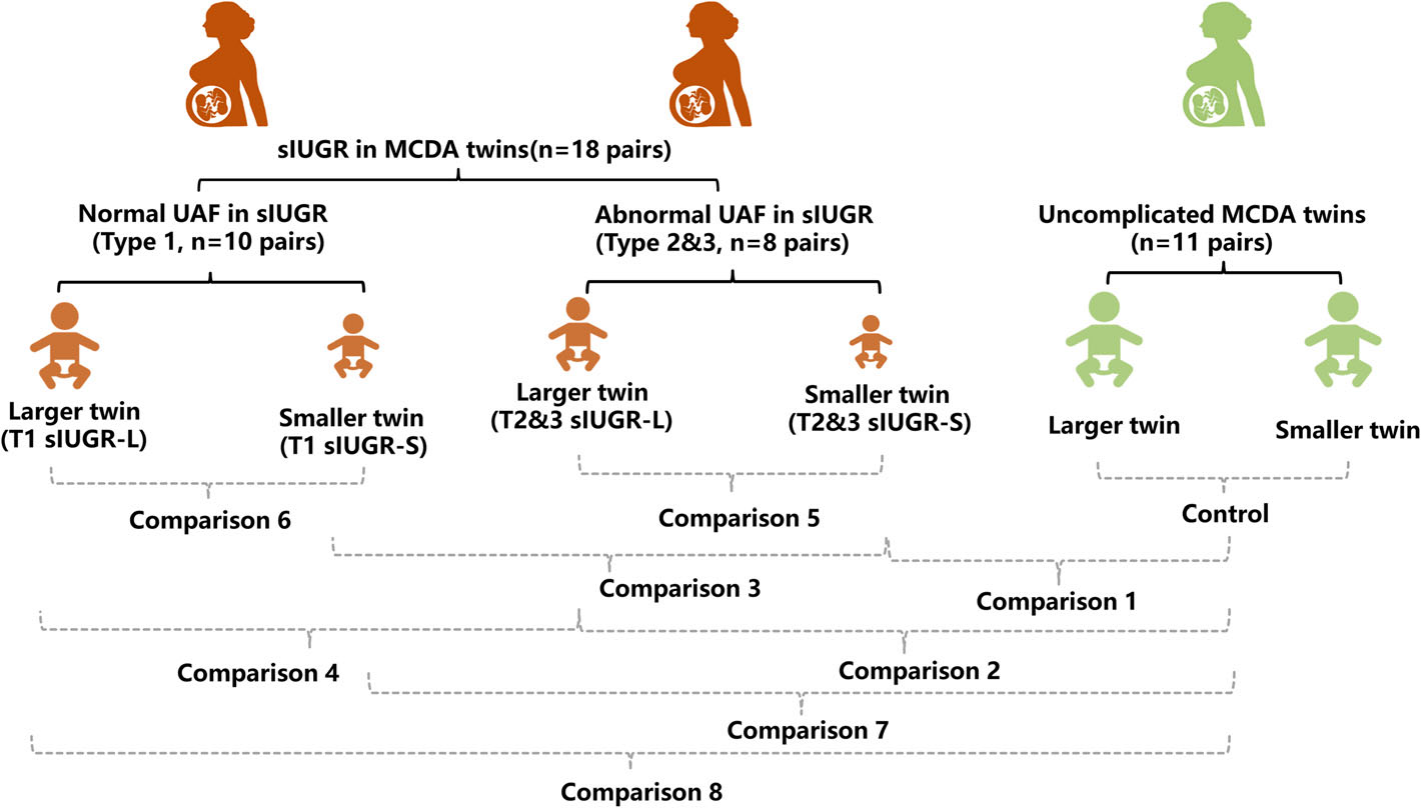
Schematic diagram of study design. 18 sIUGR MCDA pregnancies and 11 non-complicated MCDA pregnancies were included in this twin study. Among sIUGR pregnancies, 10 twin pairs were diagnosed with normal umbilical artery flow (type 1) and 8 twin pairs were abnormal UAF (types 2&3). Hairs were collected from each twin immediately after delivery for subsequent metabolomic profiling. Comparison 1 compares the metabolic disparity between T2&3 sIUGR-S and Control. Comparison 2 compares T2&3 sIUGR-L and Control. Comparison 3 compares T1 sIUGR-S and T2&3 sIUGR-S. Comparison 4 compares T1 sIUGR-L and T2&3 sIUGR-L. Comparison 5 examines T2&3 sIUGR-L and T2&3 sIUGR-S. Comparison 6 evaluates T1 sIUGR-L and T1 sIUGR-S. Comparison 7 examines T1 sIUGR-S and Control. Comparison 8 compares T1 sIUGR-L and Control. Abbreviations are as follows; sIUGR = Selective intrauterine growth restriction; MCDA = Monochorionic diamniotic; T1 = Normal umbilical artery flow; T2&3 = Abnormal umbilical artery flow; L = Larger twin; S = Smaller twin

The MCDA twin pregnancies were organized into three maternal groups: A) sIUGR pregnancy with one of the umbilical cords observed as normal UAF (type 1); B) sIUGR pregnancy with abnormal UAF (types 2&3); C) Control twin without any adverse pregnancy condition. Each pregnancy group (except control pregancy which was considered as a single group) was further divided into a larger and a smaller twin pair resulting in a total of five twin fetal groups with the following acronyms: 1) Type 1 sIUGR smaller twin (T1 sIUGR-S); 2) Type 1 sIUGR larger twin (T1 sIUGR-L); 3) Type 2 & type 3 sIUGR smaller twin (T2&3 sIUGR-S); 4) Type 2 & type 3 sIUGR larger twin (T2&3 sIUGR-L)); 5) Control twin. The unequal placental sharing, discordant velamentous cord insertion, large arterioarterial anastomoses, and abnormal umbilical artery flow of T1&2&3 sIUGR are shown in Fig. 1. Our metabolomic study was designed to differentiate metabolite differences in the neonatal hair metabolome associated with the intrauterine environmental perturbation resulting from abnormal UAF. Thus, we performed the following eight comparisons: Comparison 1 compares the smaller twins of T2&3 sIUGR and Control twins; Comparison 2 compared the larger twin of T2&3 sIUGR and Control twins. Comparison 3 compared T1 sIUGR-S and T2&3 sIUGR-S. Comparison 4 compared T1 sIUGR-L and T2&3 sIUGR-L. larger and smaller twin pairs within T2&3 sIUGR and T1 sIUGR were investigated to study the within-twin pair relationships as comparisons 5 and 6. Lastly, the smaller and larger twins of T2&3 sIUGR were compared to Control twins as comparsion 7 and 8. All eight comparisons are illustrated in Fig. 2.

### Maternal outcomes

Maternal characteristics of T1 sIUGR, T2&3 sIUGR, and normal MCDA pregnancies were listed in Supplementary Table 1. There were no statistically significant differences in age, BMI, weight gain during pregnancy, gravidity, ethnicity, delivery method, employment status during pregnancy, assisted reproduction, or karyotype test between three groups. Only gestational age at delivery was significantly different between T2&3 sIUGR and both T1 sIUGR (*p*-value = 0.001) and control MCDA pregnancies (p-value = 0.00053). This was due to the fact that sIUGR pregnancies with abnormal UAF were delivered by cesarean section prior to 32 weeks’ gestation to prevent stillbirth.

### Twin perinatal outcomes

**Fig. 3:**
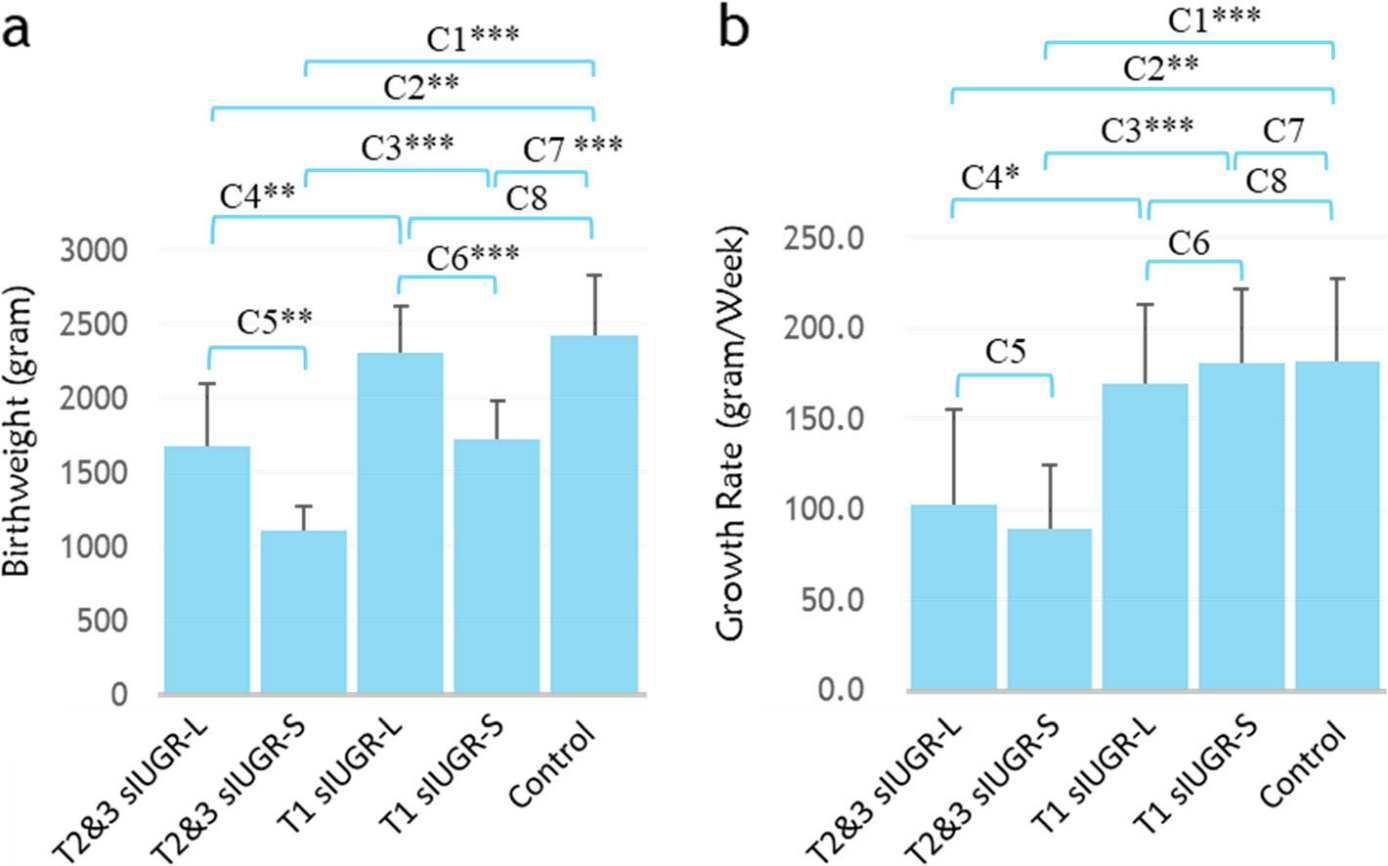
Bar graphs show the birthweight (**a**) and growth rate (**b**) of all MCDA twins. The growth rate was measured every two weeks by ultrasound after 28 gestational weeks. Statistical significance between eight comparisons (C1-C8) were determined using Student’s t-test (*p < 0.05; **p < 0.01; ***p < 0.001). List of abbreviations: MCDA = Monochorionic diamniotic; sIUGR = Selective intrauterine growth restriction; T1 = Normal umbilical artery flow; T2&3 = Abnormal umbilical artery flow; L = Larger twin; S = Smaller twin

The comparison of perinatal outcomes is described in Supplementary Table 2. No disparity in perinatal outcomes between the larger and smaller twins was observed in control MCDA twin pairs, while a significantly higher birth weight, length, abdominal circumference, and head circumference in sIUGR pregnancies was observed in the larger twins of T1 sIUGR and T2&3 sIUGR pregnancies, compared to the smaller twins in the corresponding groups. In contrast, fetal growth rate after 28 weeks’ gestation was similar between the larger and smaller sIUGR twin pairs despite birthweight disparity. The fetal growth rate was suppressed dramatically when T2&3 sIUGR twin pairs were compared to T1 siUGR and control twin pairs (Fig. 3). There were no significant differences in Apgar score at 1 min, Apgar score at 5 min, or infant sex between any of the larger and smaller co-twin comparisons.

### Comparison of the hair metabolome profiles between T1 sIUGR, T2&3 sIUGR, and control MCDA twins

**Fig. 4:**
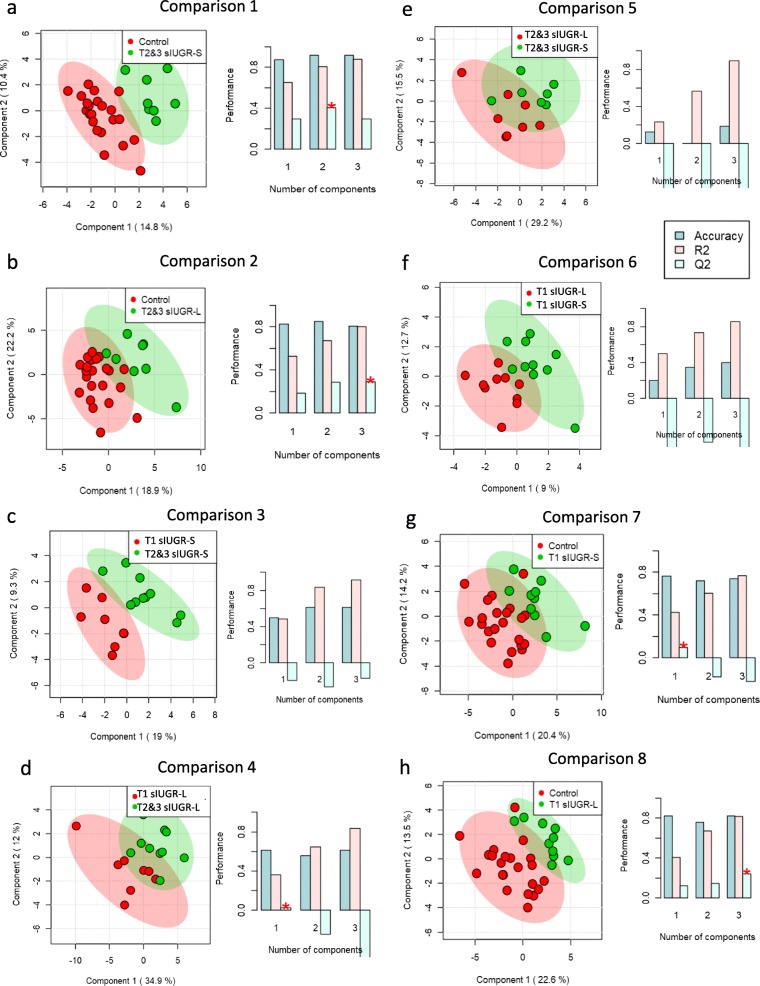
Partial least squares discriminant analysis (PLS-DA) of the hair metabolome between the eight MCDA twin comparisons, including a measure of prediction model performance (right bar graphs). List of abbreviations; sIUGR = Selective intrauterine growth restriction; T1 = Normal umbilical artery flow; T2&3 = Abnormal umbilical artery flow types; L = Larger twin; S = Smaller twin. The right bar graphics are leave-one-out cross validations (LOOCV), where R2 indicates how well the model explains the data and Q2 indicates reproducibility of the PLS-DA model

**Fig. 5:**
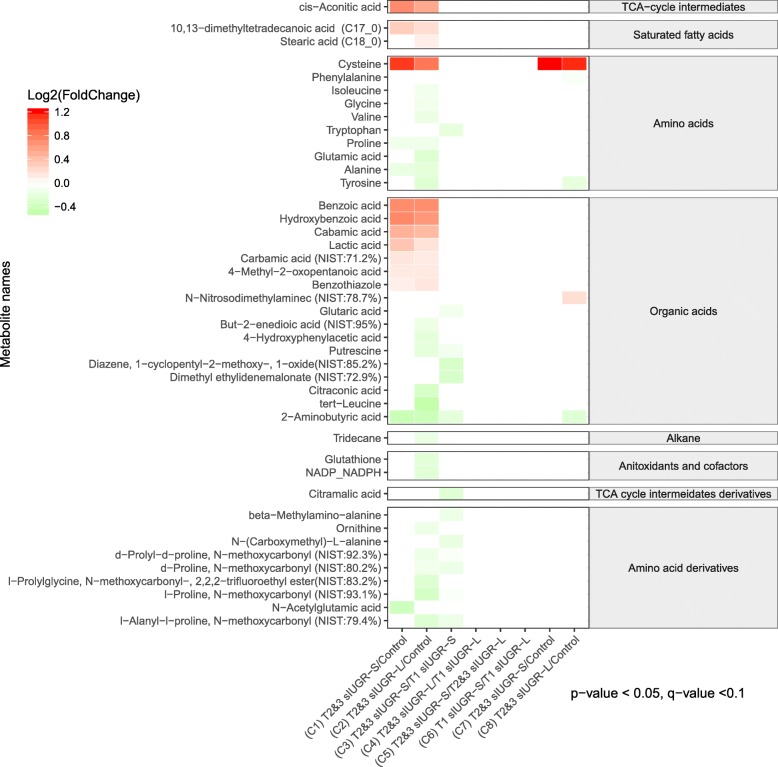
The heatmap shows the differences in the hair metabolome and associated metabolic pathways between the eight comparisons of MCDA twins. The relative concentrations of hair metabolites are illustrated via a log_2_ scale. Red color blocks represent higher metabolite levels in dividend groups than the divisor groups, whereas green color blocks represent lower metabolite levels in dividend groups than the divisor groups. Only the metabolites with a *p*-value less than 0.05 (Logistic regression adjusted for gestational age) and a q-value less than 0.01 (false discovery rate) are displayed. List of abbreviations; MCDA = Monochorionic diamniotic; sIUGR = Selective intrauterine growth restriction; T1 = Normal umbilical artery flow; T2&3 = Abnormal umbilical artery flow; L = Larger twin; S = Smaller twin

**Fig. 6:**
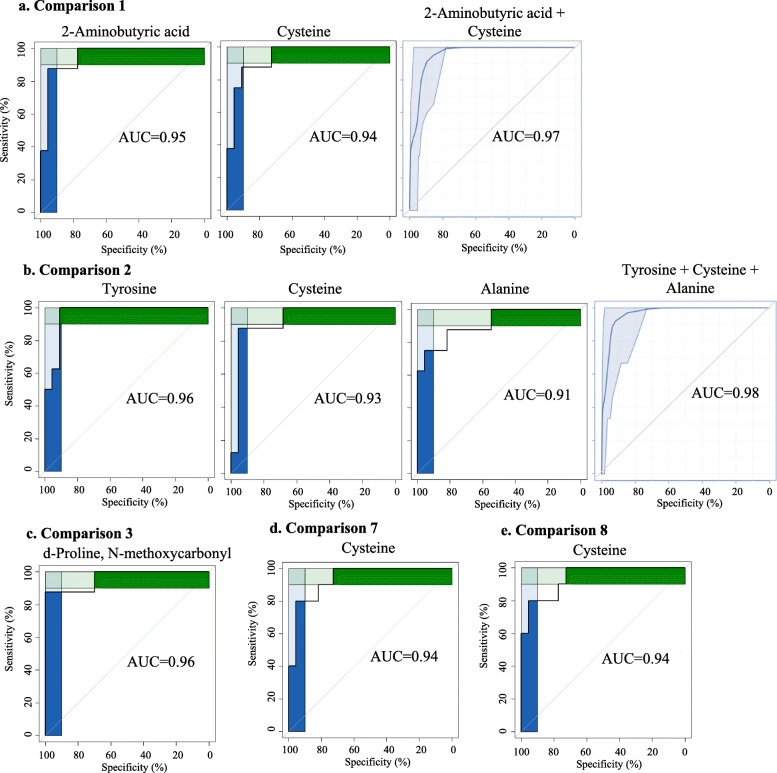
Hair metabolites with good predictive ability for the corresponding comparisons, with an area under the receiver operating characteristic (ROC) curve greater than 0.9. Pairwise comparisons are conducted between the following twin groups; **a)** Comparison 1: T2&3 sIUGR-S vs Control. The image on the right-hand side is a multivariate ROC curve combining 2-aminobutyric acid and cysteine; **b)** Comparison 2: T2&3 sIUGR-L vs Control; **c)**. Comparison 3: T2&3 sIUGR-S vs T1 sIUGR-S. The image on the right-hand side is a multivariate ROC curve combining tyrosine, cysteine, and alanine; **d)**. Comparison 7: T1 sIUGR-S vs Control; **e)**. Comparison 8: T1 sIUGR-L vs Control. List of abbreviations; sIUGR = Selective intrauterine growth restriction; L = Larger twin; S = Smaller twin; T1 = Normal umbilical artery flow; T2&3 = Abnormal umbilical artery flow

A total of 136 metabolites were identified in the twin hair samples using our in-house MCF mass spectral library and NIST library (https://www.nist.gov/nist-research-library) with the inter-assay coefficient of variation in QC samples ranging from 1.1 to 29.6% (See Supplementary Table 3). Representative metabolites are labelled on a GC-chromatogram as illuatrated in Supplementary Figure 2. The PLS-DA showed the most distinct separations and the most valid LOOCV when comparing the T2&3 sIUGR smaller and larger twins to the control group (Fig. 4a and b). Comparison 1 between T2&3 sIUGR-S and Control resulted in a PLS-DA model with high performance (Accuracy = 0.90, R2 = 0.81, Q2 = 0.48); the major components 1 and 2 accounted for 10.4 and 14.8% of variation in the metabolite levels, respectively. Meanwhile, only a minor dissimilarity was observed when comparing within larger and smaller co-twin pairs for both T2&3 sIUGR and T1 sIUGR, as illustrated in Fig. 4e & f. To remove the potential confounding effect of gestational age at delivery, adjusted logistic regressions were performed for all pairwise comparisons. The results of the logistic regression revealed 43 hair metabolites that were significantly different in concentration between the eight comparisons with *p*-value and q-value less than 0.05 and 0.1 respectively (Fig. 5). Most of the discriminating metabolites were detected in comparisons 1 and 2, where the control group was compared to both T2&3 sIUGR smaller and larger twins. Both comparisons displayed similar concentration changes in metabolite levels; one TCA cycle intermediate, one saturated fatty acid, one amino acid, and seven organic acids were found in higher levels in both T2&3 sIUGR smaller and larger twins compared to the control group, while only two amino acids and one organic acid were found in lower levels in T2&3 sIUGR twin pairs. Interestingly, cysteine is the only metabolite which appeared to be significantly reduced under four comparisons: T2&3 sIUGR twin pairs (C1, C2) and T2&3 sIUGR twin pairs (C7, C8). Meanwhile, 2-aminobutyric was significantly reduced in sIUGR twins throughout comparisons 1 to comparison 3. To further shortlist the significant metabolites that may have clinical implications, ROC curves were carried out for all metabolites that were significant across any of the eight comparisons. Figure 6 presents the following hair metabolites with an area under the ROC curve > 0.9: Comparison 1 – cysteine and 2-aminobutyric acids; Comparison 2 - three amino acids (tyrosine, cysteine, and alanine); Comparison 3 - d-proline N-methoxycarbonyl; Comparison 4 to 6 – no metabolites; Comparison 7 and 8 - cysteine only. Multivariate ROC curves were also performed using selected significant metabolites and these improved the AUC to 0.97 and 0.98 for comparisons 1 and 2 respectively.

### Correlation of hair metabolites with birthweight and growth rate, for within and between co-twin pairs

To investigate how hair metabolites correlate with birth weight and growth rate within twin pairs (comparison between the smaller and larger twins) and between twin pairs (comparison between each co-twin pairs), a GEE regression model was performed, which also accounted for the individual (twin) and shared (maternal) factors in this study. In terms of birthweight (Supplementary Figure 3), a total of 17 metabolites were significantly associated with birthweight discrepancy within the larger and smaller twin pairs. Meanwhile, 58 metabolites were significantly associated with birthweight between twin pairs. All amino acids, alkanes, antioxidants, fatty acids, and most of the amino acid derivatives were significantly different between twin pairs. With regard to fetal growth rate after 28 weeks’ gestation (Supplementary Figure 4), a total of 19 metabolites were significantly associated with growth rate disparity within the larger and smaller twin pairs, meanwhile a sum of 54 metabolites were significantly associated with the growth rate between twin pairs. The majority of hair metabolites were negatively associated with birthweight between twin pairs, while the majority of metabolites were positively associated with fetal growth rate between twin pairs.

### Predicting difference in metabolic pathway activities reflected in hair between T1 sIUGR, T2&3 sIUGR, and control MCDA twins

**Fig. 7:**
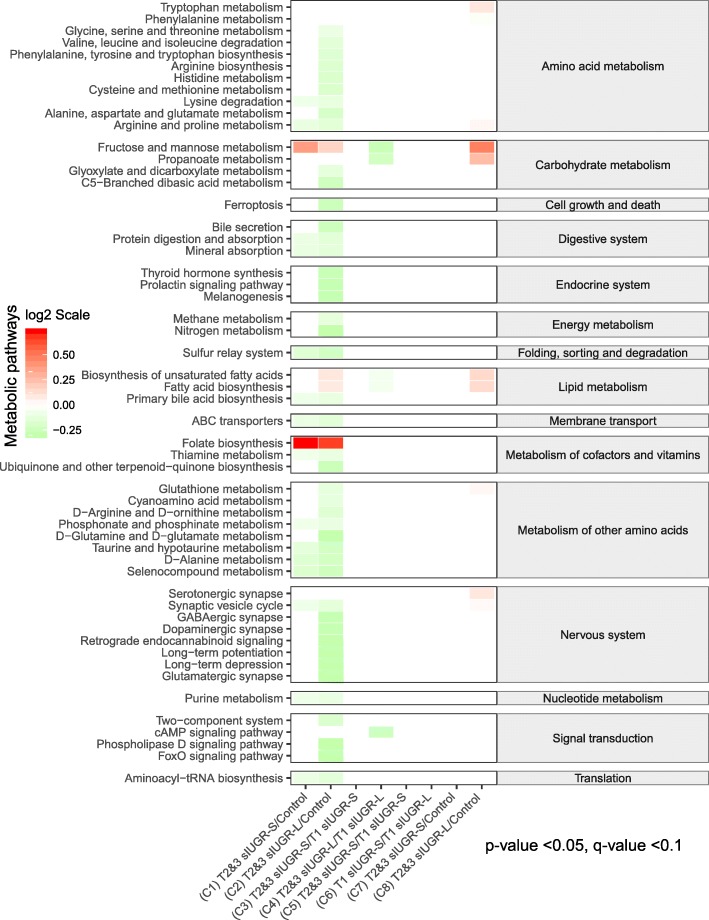
Comparing metabolic pathways in the hair metabolome associated with T1 sIUGR, T2&3 sIUGR, and control MCDA twins. The predicted metabolic activity was illustrated using log_2_ fold changes. Red color blocks represent upregulated metabolic activity in dividend groups compared to the divisor groups, whereas green color blocks represent downregulated metabolic activity in dividend groups compared to the divisor groups. Only the metabolic pathways with a significant p-value (logistic regression: *p* < 0.05) and q-value (FDR: q < 0.05) are plotted. List of abbreviations: MCDA = Monochorionic diamniotic; sIUGR = Selective intrauterine growth restriction; T1 = Normal umbilical artery flow type. T2&3 = Abnormal umbilical artery flow; L = Larger twin; S = Smaller twin

**Fig. 8:**
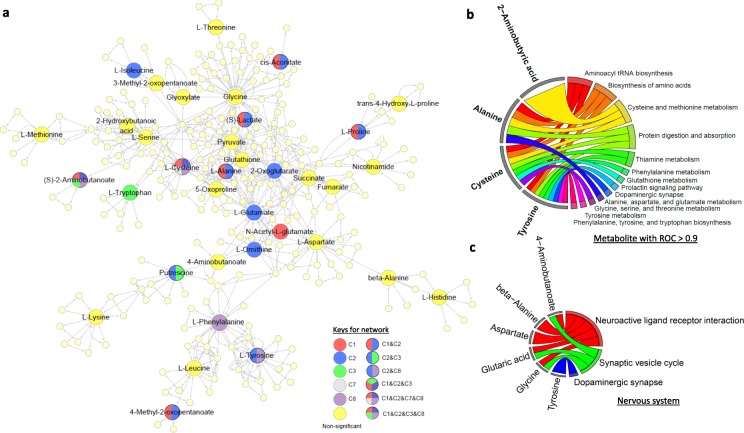
The metabolic networks represented in the hair metabolome of MCDA twins. **a** The two-dimensional network was constructed using the metabolic pathways that were significantly different between the different sIUGR clinical forms and control twins. The red circles are metabolites that were significantly different between T2&T3 sIUGR-S and Control abbreviated as C1. The blue circles are metabolites that were significantly different between T2&T3 sIUGR-L and Control abbreviated as C2. The green circles are metabolites that were significantly different between T2&T3 sIUGR-S and T1 sIUGR-S abbreviated as C3. The grey circles are metabolites that were significantly different between T1 sIUGR-S and Control abbreviated as C7. The purple circles are metabolites that were significantly different between T1 sIUGR-L and Control abbreviated as C8. Metabolites present in multiple comparisons are merged with multiple colors. The yellow circles are identified metabolites that exhibited no statistical significance. All smaller yellow circles are unidentified metabolites that were directly linked to identified metabolites. The arrowheads indicate the direction of the metabolic reactions. **b** A chord plot displays how metabolites with ROC curve > 0.9 participate in different significant metabolic pathways. **c** A chord plot displays how metabolites involved in the nervous system link to metabolic pathways

The identified hair metabolites were used to investigate the differences in metabolic activity occurring in-utero between the different twin pairs (Fig. 7). We observed that the majority of metabolic pathways were downregulated in T2&3 sIUGR-L compared to Control (comparison 2). Only a few metabolic pathways were downregulated in comparison 1 (T2&3 sIUGR-S vs Control), comparison 4 (T2&3 sIUGR-L vs T1 sIUGR-L), and comparsion 8 (T1 sIUGR-L vs Control), whereas no significant metabolic changes were detected in comparison 3 (T2&3 sIUGR-S vis T1 sIUGR-S), comparison 5 (T2&3 sIUGR-L vs T2&3 sIUGR-S), comparison 6 (T1 sIUGR-L vs T1 sIUGR-S) or comparsion 7 (T1 sIUGR-S vs Control). The significant pathways were linked to their shared metabolites and reconstructed in silico into a metabolic network based on the KEGG metabolic framework, as illustrated in Fig. 8a. 2-aminobutanoate and cysteine were the only two metabolites that were significantly different across four different twin comparisons. Protein digestion and absorption covered all the amino acids with ROC curve > 0.9 including alanine, cysteine, and tyrosine (Fig. 8b). Cysteine was involved in seven significant pathways including glutathione metabolism and amino acid metabolism. Tyrosine was the only significant metabolite with a ROC curve > 0.9 that was involved in the nervous system, namely the dopaminergic synapse pathway (Fig 8c).

### Inter- and intra-observer variations for ultrasound measurements

There was no inter-observer variation for sIUGR diagnosis and fetal growth rate because all of their ultrasound examinations were performed only by a fixed twin obstetric sonographer. Moreover, intra-observer reliability of sIUGR classifications and fetal growth rate measurements were of high agreement among the observer as demonstrated by the Kappa coefficient equal to 1 for T1 sIUGR, T2 sIUGR, and T3 sIUGR (Supplementary Table 4), as well as an excellent intra-observer correlation coefficient of 0.993, (95% CI 0.987–0.998, Supplementary Figure 5) for fetal growth rate. On the other hand, the determination of gestational age via CRL ultrasound showed that inter-observer agreement between observers A and B was 0.994 (95% CI 0.986–0.998), while the intra-observer reliability of CRL measurement was 0.998 (95% CI 0.994–0.999) and 0.997 (95% CI 0.991–0.999) for observer A and B respectively (Supplementary Figure 6). These results indicated excellent reproducibility of the CRL ultrasound measurement to determine gestational age in our institution.

## Discussion

Development of type 2 and type 3 selective intrauterine growth restriction (T2&3 sIUGR) is associated with high risk of fetal and perinatal mortality for both twins, or long-term complications following exposure to ischemia and hypoxia in the uterus, especially in the smaller twin (Miyadahira et al. 2018). At present, only a few studies have investigated the metabolomes of sIUGR twins and shown an association of the umbilical cord blood metabolome with twin birthweight disparity (Wang et al. 2018; Cosmi et al. 2013). However, birthweight is the end point of sIUGR and therefore, it is difficult to understand the pathophysiological process of sIUGR twins from diagnosis to delivery. In this study, we first observed that the metabolite profiles of neonatal hair at birth could reflect differences in fetal growth velocity. Secondly, the hair metabolome encompasses longitudinal chemical information that reflected the intrauterine metabolic differences experienced by the smaller and larger twins with T1 sIUGR and T2&3 sIUGR resulting from abnormal UAF.

The metabolite profiles of hair from MCDA twins could better distinguish fetal growth rate than birthweight. Further investigation also showed that the levels of amino acids and their derivatives were positively correlated with growth rate (Supplementary Figure 4) and, negatively correlated with birthweight (Supplementary Figure 3). The underlying rationale for these observations is postulated to be that the metabolic fingerprint the hair metabolome provides reflects the assimilation of both endogenous and exogenous compounds into the hair matrix during hair growth over months (Eastman et al. 2013; Harkey 1993). Indeed, studies have documented that neonatal hair growth is commenced around the beginning of the third trimester (Gareri and Koren 2010; Paus and Cotsarelis 1999). This period of pregnancy was also the timespan when fetal growth rate was determined using ultrasound in this study. Therefore, the hair profile is capable of recording the time-averaged influence of metabolic processes occurring during fetal growth (Skröder et al. 2017). Also, in clinical practice, twins with an estimated fetal weight less than the 10th percentile according to their gestational age or 20% birthweight discordance within twin pairs are often defined as sIUGR, however, this definition is often debated, as twin infants with clinical evidence of sIUGR can have birthweights above the 10th percentile or have less than 20% birthweight discordance (Breathnach and Malone 2012). Thus, fetal growth velocity is an ideal ultrasound index for evaluating the fetal growth potential from the time of diagnosis to birth (Hiersch and Melamed 2018). Our study provides evidence that the hair metabolome shows a similar trend with fetal growth velocity and has great potential to reflect the long-term exposure of detrimental intrauterine conditions on twin growth during the third trimester of pregnancy, when neonatal hairs begin to grow.

There was no growth rate discordance observed between the larger and smaller twin pairs for T1 sIUGR and T2&3 sIUGR. This could be due to the presence of vascular anastomoses in the MC placenta, allowing bidirectional blood-flow with low resistance through the larger diameter of arterioarterial anastomoses (Fig. 1), equalizing the growth among twin pairs (Lewi et al. 2007; Denbow et al. 2000; Hack et al. 2008b). This also means both larger and smaller twins of type 2&3 sIUGR shared the same suffering from chronic ischemia and hypoxia in the uterus. We also noted that both growth rate and birthweight of twin pairs in T2&3 sIUGR were significantly attenuated compared to T1 sIUGR and normal MCDA twin pairs. Interestingly, a majority of hair metabolites within twin pair comparisons were negatively associated with both birthweight and growth rate discordance (Supplementary Figure 1 & 2, right column). A systematic review by Inklaarl et al (2014) which included eleven articles, showed that the incidence of severe cerebral injury in MC twins is strongly associated with abnormal umbilical artery Doppler (OR 7.69; 95% CI 2.56–25.00) (Lopriore et al. 2008). Valsky et al (2010) demonstrated that larger twins are more prone to utero fetal blood transfusions via the large arterio-arterial (AA) anastomoses, resulting in a higher risk of cerebral injury following long-term ischemic hypoxia (Valsky et al. 2010; Gratacós et al. 2008). Indeed, the metabolic pathway analysis conducted in our study revealed the impairment of metabolic pathways related to the nervous system only in the larger twins of the T2&3 sIUGR twin pairs (Fig. 7). Tyrosine was the hair metabolite that showed the most promising ROC curve differentiating the larger twin of T2&3 sIUGR from control twin pairs (Fig. 6b). Tyrosine also participates in the dopaminergic synapse metabolic pathway (Fig. 8c). This highlights the potential role of tyrosine in cerebral injury. Tyrosine is an aromatic amino acid that can passively pass through the blood-brain barrier. Its role in the brain is as a precursor for biosynthesis of the neurotransmitters including dopamine, norepinephrine, and epinephrine, and functions as an important component of the autonomic nervous system. Since tyrosine is an essential amino acid, its concentration in the fetal brain is directly dependent upon maternal supply. Sanz-Corté et al (2013) demonstrated a profoundly reduced level of tyrosine in early and later IUGR neonates with signs of brain vasodilation (Sanz-Cortés et al. 2013). Abnormal levels of tyrosine in the blood of premature infants have also been reported, which is marked by impaired motor activity and intellectual deficits (Partington et al. 1971).

MCDA twin hair profiles pinpoint that insufficient umbilical artery flow is associated with the depleted supply of amino acids to the twins, contributing to sIUGR. Amino acids are not only key building constituents for the fetal body but also important regulators of metabolic pathways in fetal and placental development. Several studies have indicated that compromised placental transport and metabolism of amino acids plays an important role in the pathogenesis of fetal growth restriction (Lin et al. 2012; Lin et al. 2014; Wang et al. 2008; Pogorelova et al. 2017). Consistently, almost all metabolites discriminating T2&3 sIUGR twin pairs from Control with an area under the ROC curve > 0.9 were amino acids (Fig. 6a & b). These significant amino acids including cysteine, tyrosine, and alanine, were involved in protein/mineral absorption and in endocrine and glutathione metabolism (Fig. 8b). Cysteine is a sulfur-containing amino acid involved in protein production. It is also a precursor for the biosynthesis of essential non-protein compounds including taurine, sulfate, coenzyme A, and glutathione. Physiological cysteine levels are tightly regulated within a narrow range. The inability to maintain plasma cysteine concentration below the threshold of toxicity have been related to the risks of preterm birth, preeclampsia, and small for gestational age reported in a study of 5883 women from Norway (El-Khairy et al. 2003). It has been proposed that increased plasma cysteine levels induce endothelial dysfunction, and hypocysteinemia may cause placental vascular impairment (El-Khairy et al. 2003). This is in accordance with an increased incidence of occlusive vascular disorders confirmed by high level of cysteine (Mansoor et al. 1995; El-Khairy et al. 2001; Jacob et al. 1999). Excess cysteine is known to be catabolised in the following routes: 1) The oxidative degradation of cysteine to cysteinesulfinic acid via by cysteine dioxygenase, which in turn breaks down into taurine, sulfate, or pyruvate; 2) Cysteine catabolism for coenzyme A synthesis; 3) Utilisation of cysteine for glutathione production. Although little has been reported on the association of the first two catabolic pathways with fetal growth restriction, our findings suggest that the elevated concentration of hair cysteine levels in T1&T2&3 sIUGR twins could likely be the consequence of reduced cysteine catabolism to glutathione. Our metabolic network analysis in Fig. 8b highlighted cysteine as part of the antioxidant glutathione metabolism. Schneider et al (2015) showed that chorionic arteries from IUGR are more vulnerable to oxidative stress (Schneider et al. 2015). Herrera et al (2017) demonstrated that maternal treatment with the cysteine derivative N-acetylcysteine, a compound involved in glutathione biosynthesis, corrects the umbilical and fetal systemic vascular dysfunction in IUGR guinea pigs (Herrera et al. 2017). Thus, the regulation of cysteine levels and resulting catabolic products may be related to the maintenance of redox status and placental vasculature. Although alanine is a non-essential amino acid, it can be readily broken down and used as a major energy source. The reduction of placental transportation of alanine in IUGR has been reported in previous studies (Wu et al. 2011). The findings from our hair metabolome analysis support a growing body of evidence that sIUGR is associated with an abnormal placental-fetal transfer of amino acids, thus affecting the function of their downstream metabolic pathways.

Lastly, the hair levels of 2-aminobutyric acid could be a potential biomarker for sIUGR twins as a result of prolonged nutrient depletion. 2-aminobutyric acid was the only hair metabolite that appeared to be significantly different throughout comparisons 1 to 3 and displayed a reliable AUC (0.95) (Figs. 5 and 6). 2-aminobutyric acid is primarily produced from the breakdown of methionine, threonine, and serine. It can be commonly found in the human liver and kidney, and in most biofluids including urine, blood, and breast milk. 2-aminobutyric acid is significantly elevated in the plasma of humans with high protein diets (Haschke-Becher et al. 2016) and has been reported to reflect the body’s fuel regulation under fasting conditions (Rubio-Aliaga et al. 2011). Our results also indicated that 2-aminobutyric acid was positively associated with the growth rate of twin pairs (Fig. 8). In addition, 2-aminobutyric acid is also involved in glutathione biosynthesis (Lu 2009). Other studies have shown that oxidative stress in fetal-placental units of sIUGR is related to the dual effect of maternal undernutrition and twinning (Sales et al. 2018). Therefore, prolonged nutrient deficiency in sIUGR twins may lead to the increased 2-aminobutyric acid levels observed in the hair metabolome, as well as reduced antioxidant capability.

## Conclusions

This is the first study to map the neonatal hair metabolome to fetal growth velocity and birthweight of sIUGR twins with abnormal UAF. The neonatal hair profile revealed a phenotype of abnormal UAF in twins which included a positive relationship with fetal growth velocity among T1&2&3 sIUGR twin pairs. The metabolites including 2-aminobutyric acid, cysteine, and tyrosine were associated with growth retardation and antioxidant capability, emphasising the ability of hair to reflect the intrauterine condition as well as an avenue to diagnose the severity of fetal growth restriction. It should be noted that the relationship between the hair metabolome and fetal growth velocity is only applicable for the third trimester of pregnancy, when neonatal hair growth has been initiated. A clinical follow-up study on twins is required to confirm the potential use of the neonatal hair profile to predict whether the T1/T2&3 sIUGR twins will return to normal growth potential after delivery, and to what degree cognitive development is affected in the infant. If the early detection of adverse outcomes can be achieved then appropriate therapeutic interventions for sIUGR twins could be tailored, minimizing the effects of fetal growth restriction and improving growth, health, and well-being of sIUGR twins. Lastly, due to the rarity of sIUGR twin pregnancies and a high rate of intrauterine fetal death, only a small sample size of 18 sIUGR pregnancies with two live fetuses were included in this study. A clinical follow-up study with larger recruitment of twins is required to confirm the potential use of the newborn infant hair profile to study the metabolic perturbations of sIUGR twins.

## Supplementary information

**Additional file 1: Supplementary Table 1**. Participant Characteristics. **Supplementary Table 2**. Clinical outcomes of different twin groups. **Supplementary Table 3** Identified metabolites. **Supplementary Table 4**. Kappa value to measure the intra-observer agreement on the classifications of T1 sIUGR, T2 sIUGR, and T3 sIUGR. **Supplementary Figure 1**. Flowchart of study MCDA twin pregnancies. **Supplementary Figure 2**. Representative total ion chromatogram (TIC) of the neonatal hair metabolome. **Supplementary Figure 3**. A generalized estimating equation to correlate the hair metabolites associated with birthweight discordance within and between MCDA twin pairs between T1 sIUGR, T2&3 sIUGR, and control twins. **Supplementary Figure 4**. A generalized estimating equation to correlate the hair metabolites associated with the growth rate discordance within and between MCDA twin pairs between T1 sIUGR, T2&3 sIUGR, and control twins. **Supplementary Figure 5**. Intra-observer variability in the measurement of fetal growth rate. **Supplementary Figure 6.** The inter-observer variability (a), intra-observer A variability (b) and intra-observer B variability (c) for determining gestational age. **Supplementary Figure 7**. PCA analysis of hair metabolite profile (*n* = 3 per group) stored at various temperatures over six months.

## Data Availability

The datasets used and/or analysed during the current study are available from the corresponding author on reasonable request.

## Abbreviations

AC: Abdominal circumference
BPD: Biparietal diameter
DC: Dichorionic
EFW: Estimated fetal weight
FDR: False discovery rate
FL: Femur length
GC-MS: Gas chromatography-mass spectrometry
GDM: Gestational diabetes mellitus
GEE: Generalized estimating eq.
HC: Head circumference
ISUOG: International Society of Ultrasound in Obstetrics and Gynecology
LOOCV: Leave-one-out cross validation
MC: Monochorionic
MCDA: Monochorionic diamniotic
PAPi: Pathway Activity Profiling
PLS-DA: Partial least squares discriminant analysis
QC: Quality control
ROC: Receiver operating characteristic
sIUGR: Selective intrauterine growth restriction
T2&3: Types 2 and 3
UAF: Umbilical artery flow
