# Supplementary material for: Neonatal hair profiling reveals a metabolic phenotype of monochorionic twins with selective intrauterine growth restriction and abnormal umbilical artery flow
Source: Mol Med. 2020 May 1;26:37. doi: 10.1186/s10020-020-00160-8 (PMC7193362; doi:10.1186/s10020-020-00160-8)
Supplement: Supplementary file 1 — Additional file 1: Supplementary Table 1. Participant Characteristics. Supplementary Table 2. Clinical outcomes of different twin groups. Supplementary Table 3 Identified metabolites. Supplementary Table 4. Kappa value to measure the intra-observer agreement on the classifications of T1 sIUGR, T2 sIUGR, and T3 sIUGR. Supplementary Figure 1. Flowchart of study MCDA twin pregnancies. Supplementary Figure 2. Representative total ion chromatogram (TIC) of the neonatal hair metabolome. Supplementary Figure 3. A generalized estimating equation to correlate the hair metabolites associated with birthweight discordance within and between MCDA twin pairs between T1 sIUGR, T2&3 sIUGR, and control twins. Supplementary Figure 4. A generalized estimating equation to correlate the hair metabolites associated with the growth rate discordance within and between MCDA twin pairs between T1 sIUGR, T2&3 sIUGR, and control twins. Supplementary Figure 5. Intra-observer variability in the measurement of fetal growth rate. Supplementary Figure 6. The inter-observer variability (a), intra-observer A variability (b) and intra-observer B variability (c) for determining gestational age. Supplementary Figure 7. PCA analysis of hair metabolite profile (n = 3 per group) stored at various temperatures over six months. [file 10020_2020_160_MOESM1_ESM.pdf]

Neonatal hair profiling reveals a metabolic phenotype of monochorionic twins with selective intrauterine growth restriction and abnormal umbilical artery flow

Jing Yang, Yuan Wei, Hongbo Qi, Nanlin Yin, Yang Yang, Zailing Li, Lili Xu, Xueju Wang, Pengbto Yuan, Luyao Li, Ting-Li Han, Yangyu Zhao

Supplementary Table 1. Participant Characteristics

	**Type 2&3 sIUGR (n=8)[a]**	**Type 1 sIUGR (n=10)[b]**	**Control (n=11)[c]**	**p-values a vs b**	**p-values a vs c**
Maternal age (years)	28.3 ± 3.3	31.0 ± 3.4	30.5 ± 2.7	0.15[1]	0.14[1]
Maternal BMI (kg/m^2)	22.8 ± 3.3	21.7 ± 3.3	21.8 ± 5.7	0.26[1]	0.60[1]
Gestational age at delivery (weeks)	30 (29.8, 30.5)	35 (33.5, 35)	36 (35.5, 37)	0.0011[2]**	0.00053[2]***
Umbilical blood pH	7.3 ± 0.08	7.4 ± 0.05	7.4 ± 0.04	0.12[1]	0.12[1]
Weight gain during pregnancy (kg)	12.7 ± 4.1	15 ± 4.3	18.2 ± 6.9	0.32[1]	0.050[1]
Primigravida				1[3]	1[3]
Yes	5 (62.5%)	6 (60.0%)	8 (72.7%)		
No	3 (37.5%)	4 (40.0%)	3 (27.2%)		
Han ethnicity				1[4]	1[4]
Yes	8 (100%)	10 (100%)	11 (100%)		
No	0	0	0		
Delivery method				1[3]	0.43[4]
Cesarean	7 (87.5%)	9 (90.0%)	11 (100.0%)		
Vaginal	1 (12.5%)	1 (10.0%)	0 (0.0%)		
Employment				1[3]	0.83[3]
Yes	7(87.5%)	9 (90.0%)	8 (72.7%)		
No	1(12.5%)	1 (10.0%)	3 (27.3%)		
IVF-ET/ Natural conception				1[4]	0.60[4]
IVF-ET	0 (0.0%)	1(10.0%)	2 (18.2%)		
Natural conception	8 (100.0%)	9(90.0%)	9 (81.8%)		
Karyotype test				1[4]	0.60[4]
NIPT	8 (100.0%)	10 (100.0%)	9 (81.8%)		
Amniocentesis	0 (0.0%)	0 (0.0%)	2 (18.2%)		

[1]Student's T-test; [2]Mann-whitney U test; [3]Chi-square test; [4]Fisher's exact test; *p<0.05; **p<0.01; ***p<0.001; IVF-ET = *In Vitro* fertilization-embryo transfer; NIPT = Non-invasive prenatal testing.

Supplementary Table 2. Clinical outcomes of different twin groups

	Type 2& 3 sIUGR twins			Type 1 sIUGR twins			Control twins		
	T1(n=8)	T2(n=8)	p-value	T1(n=10)	T2(n=10)	p-value	T1(n=11)	T2(n=11)	p-value
**Birth weight (g)**	1555 (1293, 2125)	1055 (988, 1198)	0.0054[2]**	2310 ± 308	1729 ± 246	0.00022[1]***	2421 ± 405	2272 ± 418	0.41[1]
**Growth Rate (g/week) after 28 weeks**	103 ± 51	89 ± 35	0.55[1]	169 ± 44	181 ± 41	0.53[1]	181 ± 46	170 ± 33	0.53[1]
**Height (cm)**	39.1 ± 2.2	36.25 ± 2.4	0.028[1]*	44.8 ± 2.1	42 ± 2.4	0.012[1]*	46 (44, 46)	44 (43, 46)	0.38[2]
**AC (cm)**	26.5 (25.5, 32)	23.5 (22, 26)	0.047[2]*	30.7 ± 2.0	27.6 ± 1.6	0.0014[1]**	31 (30, 32)	30 (29.5, 31)	0.21[2]
**HC (cm)**	31.3 (18.2, 33)	26.5 (25.8, 27.3)	0.019[2]*	32.5 ± 1.4	30.1 ± 1.4	0.0016[1]**	33 (32.5, 34)	33 (31.5, 33)	0.14[2]
**Apgar score at 1 min**	7.25 ± 2.7	7.4 ± 1.9	0.92[1]	10 (10, 10)	10 (9, 10)	0.61[2]	10 (10, 10)	10 (9,10)	0.53[2]
**Apgar score at 5 min**	9 (8, 10)	9 ± (9, 10)	0.62[1]	10 (10, 10)	10 (10, 10)	0.58[2]	10 (10, 10)	10 (10, 10)	0.58[2]
**Gender**			1[3]			1[3]			1[3]
Male	5 (62.5%)	5 (62.5%)		5 (50%)	5 (50%)		6 (54.5%)	6 (54.5%)	
Female	3 (37.5%)	3 (37.5%)		5 (50%)	5 (50%)		5 (45.5%)	5 (45.5%)	

[1]Student's T-test; [2]Mann-whitney U test; [3]Chi-square test; *p<0.05; **p<0.01; ***p<0.001; sIUGR = Selective intrauterine growth restriction; AC = Abdominal circumference; HC = Head circumference; T1= Larger twin; T2=Smaller twin.

Supplementary Table 3 Identified metabolites

Metabolite names	Identifications	% library Match	CV (%)
Proline	Confidently identified	100	2.1
Dodecane	Confidently identified	99	3.6
Glutamic acid	Confidently identified	99	13.9
Glycine	Confidently identified	99	1.6
Lactic acid	Confidently identified	99	11.9
Ornithine	Confidently identified	99	7.6
Phenylalanine	Confidently identified	99	1.8
Pyruvic acid	Confidently identified	99	26.6
Valine	Confidently identified	99	1.3
10,13-dimethyltetradecanoic acid (C17_0)	Confidently identified	98	11.4
Aspartic acid	Confidently identified	98	4.5
Glutathione	Confidently identified	98	13.3
Isoleucine	Confidently identified	98	2.5
Benzoic acid	Confidently identified	97	10.9
Leucine	Confidently identified	97	1.7
Threonine	Confidently identified	97	7.0
Alanine	Confidently identified	96	1.6
Citraconic acid	Confidently identified	96	10.5
Pyroglutamic acid	Confidently identified	96	5.9
4-Aminobutyric acid (GABA)	Confidently identified	95	13.8
Serine	Confidently identified	95	16.2
Tyrosine	Confidently identified	95	4.2
2-Oxoglutaric acid	Confidently identified	93	7.1
1-Aminocyclopropane-1-carboxylic acid	Confidently identified	92	10.3
2,4-Di-tert-butylphenol	Confidently identified	92	7.8
3-Methyl-2-oxopentanoic acid	Confidently identified	92	14.5
Fumaric acid	Confidently identified	92	9.9
Succinic acid	Confidently identified	92	9.0
2-Aminobutyric acid	Confidently identified	91	2.8
Creatinine	Confidently identified	91	11.4
Myristic acid (C14_0)	Confidently identified	91	7.8
DBP	Confidently identified	90	7.5
NADP_NADPH	Confidently identified	90	12.4
Salicylic acid	Confidently identified	90	8.2
4-Methyl-2-oxopentanoic acid	Confidently identified	89	8.2
Citramalic acid	Confidently identified	89	3.8
Cysteine	Confidently identified	89	26.4
Dipicolinic acid	Confidently identified	89	15.7
Levulinic acid	Confidently identified	89	7.7
Methionine	Confidently identified	89	8.0
trans-Vaccenic acid	Confidently identified	89	6.0
Glutaric acid	Confidently identified	88	6.6
Dimethyl aminomalonic acid	Confidently identified	87	9.9
2-Hydroxyglutaramic acid	Confidently identified	87	4.8
3-(-2-Thienyl)-D-alanine	Confidently identified	87	1.1
Glyoxylic acid	Confidently identified	87	6.6
Malic acid	Confidently identified	87	62.2
Adipic acid	Confidently identified	85	8.2

Malonic acid	Confidently identified	85	9.3
Tryptophan	Confidently identified	85	17.4
Azelaic acid	Confidently identified	84	6.5
Heneicosanoic acid (C21_0)	Confidently identified	84	19.4
Tricosane	Confidently identified	84	4.4
DL-gamma-methyl-ketoglutaramate	Confidently identified	83	2.8
Stearic acid (C18_0)	Confidently identified	83	11.3
Cabamic acid	Confidently identified	82	12.2
Pentadecane	Confidently identified	82	2.6
Tridecane	Confidently identified	82	4.4
N-Acetylglutamic acid	Confidently identified	81	7.7
tert-Leucine	Confidently identified	81	13.5
2-Hydroxybutyric acid	Confidently identified	80	13.8
16.7644 min N-(Carboxymethyl)-L-alanine	Confidently identified	79	6.4
Caprinoic acid	Confidently identified	79	5.0
Heptadecane	Confidently identified	78	7.6
Lysine	Confidently identified	78	10.6
Norvaline	Confidently identified	78	6.3
Octanoic acid (C8_0)	Confidently identified	78	5.4
4-Hydroxyphenylacetic acid	Confidently identified	77	6.0
beta-Methylamino-alanine (BMAA)	Confidently identified	75	4.0
Nicotinamide	Confidently identified	75	25.3
Nicotinic acid	Confidently identified	74	11.7
cis-Aconitic acid	Confidently identified	73	7.4
beta-Alanine	Confidently identified	72	12.7
trans-4-Hydroxyproline	Confidently identified	72	29.6
Benzothiazole	Confidently identified	71	9.0
Hydroxybenzoic acid	Confidently identified	66	7.2
Histidine	Confidently identified	62	8.7
Dodecanoic acid (C12_0)	Confidently identified	61	5.8
Putrescine	Confidently identified	68	3.0
Ethane, hexachloro-(NIST:95.7%)	Putatively identified	99	2.5
Methyl 2-ethoxyacetate (NIST:69.8%)	Putatively identified	98	13.9
Propanedioic acid, (2-methyl-2-propenyl)-, dimethyl ester (NIST:61.5%)	Putatively identified	98	4.6
2-Oxomalonic acid, methylhydrazone, dimethyl ester(NIST:60.8%)	Putatively identified	98	9.9
Bis(2-ethylhexyl) phthalate (NIST:93.2%)	Putatively identified	97	10.3
l-Prolylglycine, N-methoxycarbonyl-, 2,2,2-trifluoroethyl ester (NIST:83.2%)	Putatively identified	96	5.8
Cyclopentane, 1,2,4-trimethyl-(NIST:73.4%)	Putatively identified	95	2.9
But-2-enedioic acid, dimethyl ester (NIST:95%)	Putatively identified	95	8.8
Cyclononasiloxane, octadecamethyl-(NIST:65.9%)	Putatively identified	94	18.8
Cyclopentasiloxane, decamethyl-(NIST:94.4%)	Putatively identified	94	29.1
Cyclohexanol, 2-amino-, cis-(NIST:69.3%)	Putatively identified	91	5.0
Pentadecane (NIST:87.6%)	Putatively identified	91	9.1
Dimethyl ethylidenemalonate (NIST:72.9%)	Putatively identified	91	6.4
d-Prolyl-d-proline, N-methoxycarbonyl-, methyl ester(NIST:92.3%)	Putatively identified	90	1.6
N-Nitrosodimethylamine (NIST:78.7%)	Putatively identified	88	6.9
l-Proline, N-methoxycarbonyl-, octyl ester (NIST:93.1%)	Putatively identified	88	9.3
Cycloheptasiloxane, tetradecamethyl-(NIST:91.4%)	Putatively identified	87	19.2
l-Alanyl-l-proline, N-methoxycarbonyl-, methyl ester (NIST:79.4%)	Putatively identified	85	3.2

l-Leucine, N-methyl-N-(2-methoxyethoxycarbonyl)-, pentyl ester (NIST:68%)	Putatively identified	85	12.4
d-Proline, N-methoxycarbonyl-, methyl ester (NIST:85.5%)	Putatively identified	82	2.4
Uracil (NIST:79.9%)	Putatively identified	79	19.1
(2R,5S)-2-Butyl-5-propylpyrrolidine (NIST:64.9%)	Putatively identified	78	6.6
Methyl tetradecanoate (NIST;64.6%)	Putatively identified	77	19.2
Dithiocarbamic acid, N, N-dimethyl-, S-dimethylamino ester (NIST:71.3%)	Putatively identified	76	4.6
2,4-Imidazolidinedione, 5-methyl-(NIST:85.4%)	Putatively identified	76	20.7
Butanedioyl dihydrazide (NIST:73.9%)	Putatively identified	72	10.7
Diazene, 1-cyclopentyl-2-methoxy-, 1-oxide (NIST:85.2%)	Putatively identified	71	13.4
Butanoic acid, -[(2-methoxy-1,2-dioxoethyl) hydrazono] - (NIST:69.2%)	Putatively identified	71	5.4
4-Pyridinecarboxaldehyde (NIST:91.1%)	Putatively identified	69	5.3
l-Proline, N-methoxycarbonyl-, hexadecyl ester (NIST:75.9%)	Putatively identified	66	8.4
d-Proline, N-methoxycarbonyl-, heptyl ester (NIST:80.2%)	Putatively identified	65	4.9
4-Aminofurazan-3-carboxylic acid, 3,3-dimethyl-2-oxobutyl ester (NIST:61.7%)	Putatively identified	60	5.8
Naphtho[1,2-b] furan-2,6(3H,4H)-dione, 3a,5,5a,9,9a,9b-hexahydro-9-hydroxy-3,5a,9-trimethyl-(NIST:66.7%)	Putatively identified	59	22.2
2-Phenylacetic acid, 2,2,2-trifluoroethyl ester (NIST:80.7%)	Putatively identified	58	6.6
Carbamic acid, methyl ester (NIST:71.2%)	Putatively identified	55	6.2
1,3-Diethoxybenzene (NIST:60.9%)	Putatively identified	55	4.8
Glutaric acid, di(3-heptyl) ester (NIST:63.9%)	Putatively identified	53	2.5
Unknown 115(100) 118(95.4) 86(69.3)	Unknown		8.5
Unknown 082(100) 142(56.8) 110(42.6)	Unknown		10.6
Unknown 252(100) 296(84.8) 236(47.6)	Unknown		6.4
Unknown 114(100) 59(61.6) 146(33.7)	Unknown		22.6
Unknown 086(100) 59(56.6) 128(39.3)	Unknown		9.9
Unknown 174(100) 70(96.8) 114(77.2)	Unknown		13.5
Unknown 115(100) 59(65.5) 189(50.1)	Unknown		7.5
Unknown 128(100) 139(21.1) 42(19.1)	Unknown		9.6
Unknown 088(100) 43(72.8) 147(38.1)	Unknown		13.7
Unknown 071(100) 59(41.6) 175(38.6)	Unknown		6.5
Unknown 125(100) 184(90.5) 96(54.3)	Unknown		8.9
Unknown 130(100) 70(47.4) 98(19.7)	Unknown		3.4
Unknown 160(100) 59(43.4) 116(22.7)	Unknown		10.7
Unknown 232(100) 128(72.0) 156(51.0)	Unknown		9.8
Unknown 144(100) 88(66.2) 70(62.9)	Unknown		4.0
Unknown 088(100) 44(30.3) 59(19.6)	Unknown		11.7
Unknown 059(100) 57(69.6) 71(61.8)	Unknown		5.6
Unknown 084(100) 126(40.0) 43(23.4)	Unknown		8.3
Unknown 091(100) 150(35.5) 65(11.2)	Unknown		15.3

CV: Coefficient of variation

Supplementary Table 4. Kappa value to measure the intra-observer agreement on the classifications of T1 sIUGR, T2 sIUGR, and T3 sIUGR

		Value	Asmp. Std. Error[a]	Approx. T[b]	Approx. Sig.
Measurement of agreement	Kappa	1.00	.000	7.066	.000
N of valid case		25			

[A] Not assuming the null hypothesis
[b] Using the asymptotic standard error assuming the null hypothesis

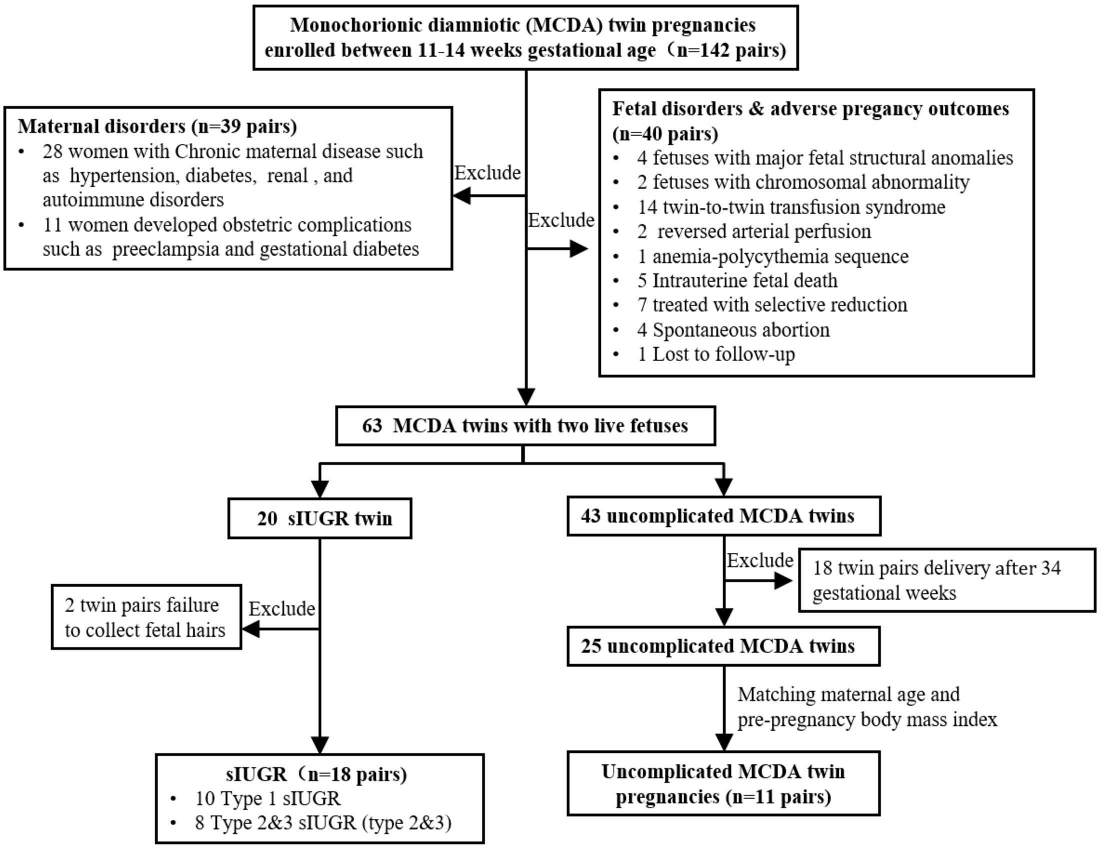

Supplementary Figure 1. Flowchart of study MCDA twin pregnancies

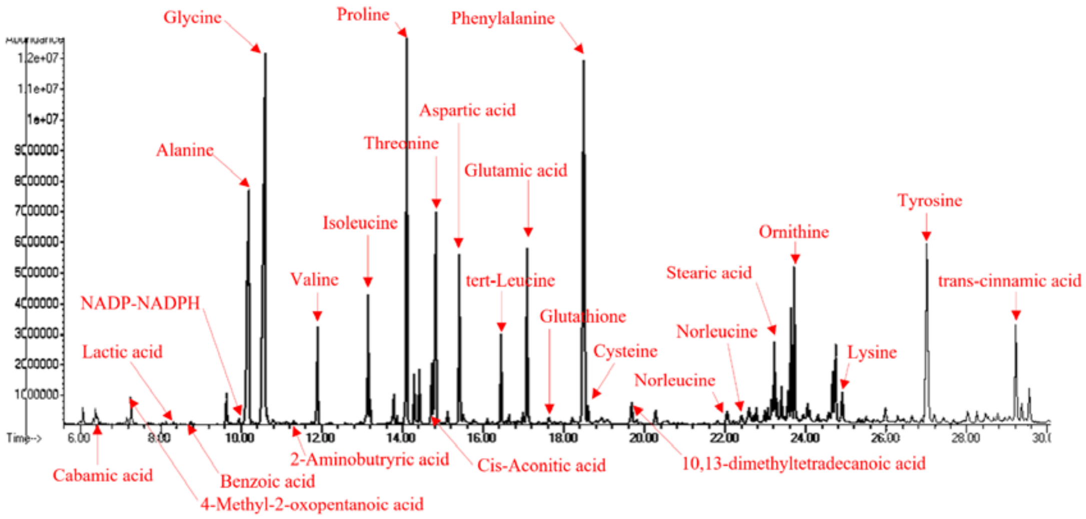

Supplementary Figure 2. Representative total ion chromatogram (TIC) of the neonatal hair metabolome.

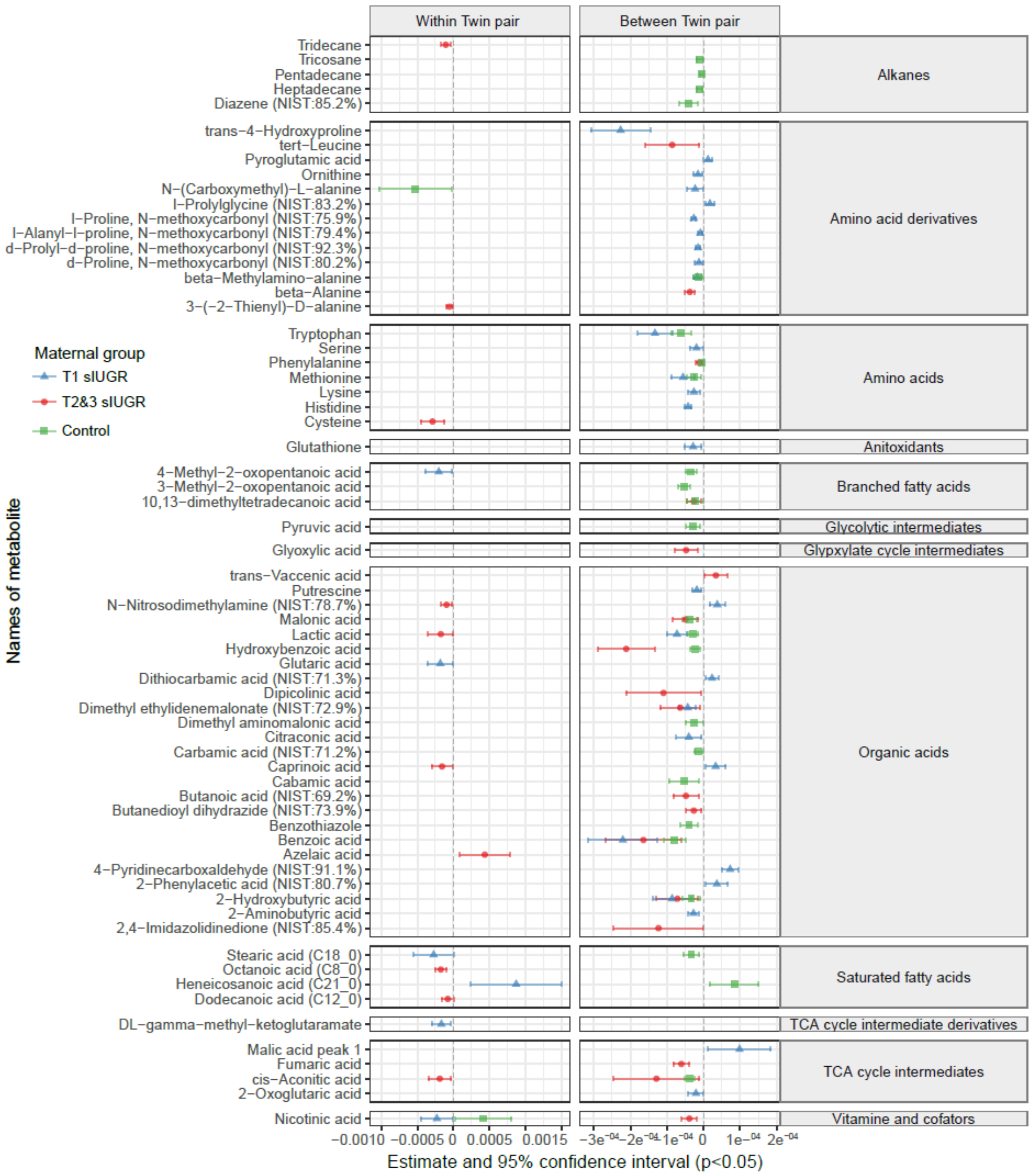

Supplementary Figure 3. A generalized estimating equation to correlate the hair metabolites associated with birthweight discordance within and between MCDA twin pairs between T1 sIUGR, T2&3 sIUGR, and control twins. Blue lines, red lines, and green lines show the 95% estimated confidence intervals for the correlation of hair metabolites with weight discordance in T1 sIUGR twins, T2&3 sIUGR twins, and control MCDA twins, respectively. The left column represents the between-twin pair regression model calculated on average twin pairs birth weight, whilst the right column shows the within-twin pair regression model calculated on the difference in birth weight between the twin pair. The middle dotted line in each column means a slope coefficient of zero; metabolites on the right of the dotted line are positively associated with birthweight discordances between/within twin pairs, while metabolites toward the left of the dotted line are negatively associated with birthweight discordances between/within twin pairs. Greater the distance from the dotted line indicates a greater change in hair metabolite level in response to a unit of change in birthweight. Hair metabolites are grouped based on their biochemical roles and only the metabolites with p-values < 0.05 are displayed.

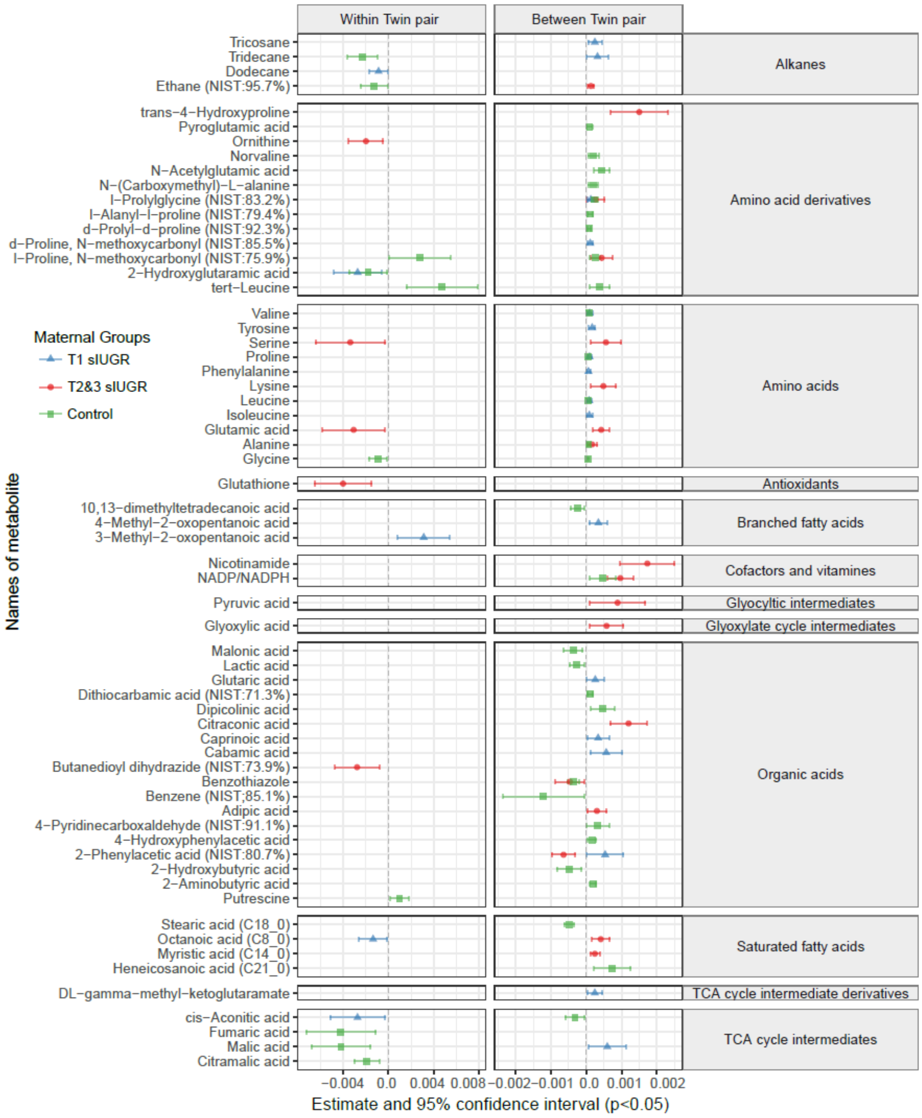

Supplementary Figure 4. A generalized estimating equation to correlate the hair metabolites associated with the growth rate discordance within and between MCDA twin pairs between T1 sIUGR, T2&3 sIUGR, and control twins. Blue lines, red lines, and green lines show the 95% estimated confidence intervals for the correlation of hair metabolites with growth rate discordance after 28 gestational weeks in T1 sIUGR twins, T2&3 sIUGR twins, and control MCDA twins, respectively. The left column represents the between-twin pair regression model calculated on average twin pairs growth rate, whilst the right column represents the within-twin pair regression model calculated on the difference in growth rate between the twin pair. The middle dotted line in each column means a slope coefficient of zero; metabolites on the right of the dotted line are positively associated with growth rate discordance between/within twin pairs, while metabolites toward the left of the dotted line are negatively associated with growth rate changes between/within twin pairs. Greater the distance from the dotted line indicates a greater change in hair metabolite level in response to a unit of change in fetal growth rate. Hair metabolites are grouped based on their biochemical roles and only the metabolites with p-values < 0.05 are displayed.

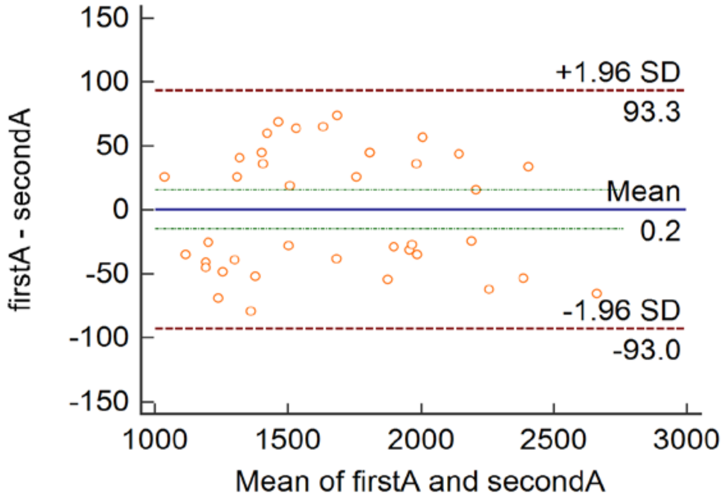

Supplementary Figure 5. Intra-observer variability in the measurement of fetal growth rate. The proportional difference from the mean for the estimated fetal weight (EFW) in 20 twin pairs measured by ultrasound between 28-36 gestational weeks by the same sonographer twice. The upper and lower dotted lines indicate the 2.5th and 97.5th percentiles for limits of agreement. The middle blue line is the mean. The intraclass correlation coefficient with the 95% CI of the fetal growth rate is 0.993 (95% CI 0.987–0.998) for this observer.

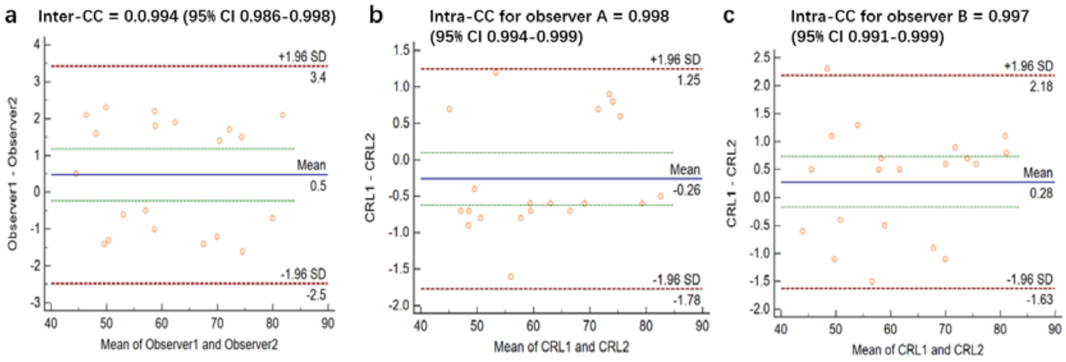

Supplementary Figure 6. The inter-observer variability (a), intra-observer A variability (b) and intra-observer B variability (c) for determining gestational age. The proportional difference from the mean for the crown–rump lengths (CRL) of 20 larger twins were performed by two independent registered sonographers (observer A and B). The upper and lower dot lines indicate the 2.5th and 97.5th percentiles for limits of agreement. The middle blue line is the mean.

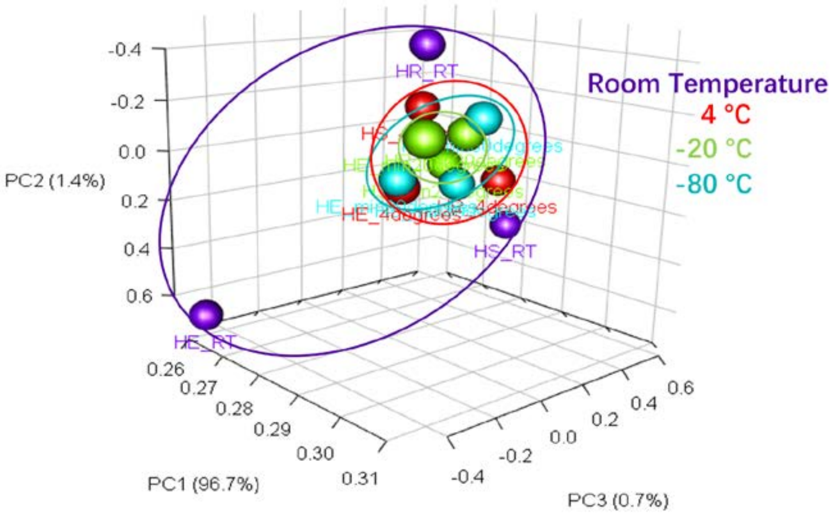

Supplementary Figure 7. PCA analysis of hair metabolite profile (n=3 per group) stored at various temperatures over six months.
